# Comparison of pregnancy complications in unintended and intended pregnancy: A prospective follow-up study

**DOI:** 10.37796/2211-8039.1192

**Published:** 2021-12-01

**Authors:** Mitra Eftekhariyazdi, Malihe Mehrbakhsh, Mahboubeh Neamatshahi, Manijeh Yousefi Moghadam

**Affiliations:** a Department of Obstetrics and Gynecology, School of Medicine, Sabzevar University of Medical Sciences, Sabzevar, Iran; b Student Research Committee, School of Medicine, Sabzevar University of Medical Sciences, Sabzevar, Iran; c Department of Community Medicine, School of Medicine, Sabzevar University of Medical Sciences, Sabzevar, Iran; d epartment of Anesthesiology, Faculty of Medicine, Sabzevar University of Medical Sciences, Sabzevar, Iran

**Keywords:** Low birth weight, Preeclampsia, Preterm labor, Unintended pregnancy

## Abstract

**Background and objectives:**

Unintended pregnancy, as a pregnancy that is mistimed, unplanned or unwanted at the time of conception, is a common experience worldwide that puts mothers at risk for mental stress and its pregnancy complications. The aim of this study was to compare three common pregnancy complications, including preeclampsia, preterm labor, and low birth weight, between unintended and intended pregnancies in Sabzevar, northeast Iran in 2019.

**Materials and methods:**

This prospective follow-up study was conducted on 200 pregnant women (100 intended and 100 unintended pregnancies) who were between 18 and 35 years old and were referred for delivery to Shahidan Mobini Hospital, Sabzevar, Iran. Data were collected using a questionnaire and the subjects were recruited based on inclusion and exclusion criteria. Preeclampsia, preterm labor, and low birth weight were recorded after delivery and were statistically analyzed using the statistical package for social sciences (SPSS software) version 22 at the statistical significance of <0.05.

**Results:**

The mean age and gravidity was significantly higher in the unintended pregnancy group compared to intended pregnancy group. The most common complication overall was low birth weight (25% of unintended and 16% of the intended pregnancies) followed by preterm labor (12% of unintended and 11% of intended pregnancies) and preeclampsia (5% of unintended and 1% of intended pregnancies). No significant relationship was found between the time of delivery and type of pregnancy (P = 0.50).

**Conclusion:**

The findings of this study indicated that unintended pregnancy can be a risk factor for pregnancy complications including preeclampsia and low birth weight and that sophisticated monitoring should be performed for better management of these complications.

## 1. Introduction

As the parents increasingly decide to have smaller families, they are more trying to control the timing and number of their births [1–5]. While contraceptive methods are not hundred percent successful in preventing pregnancies, unintended pregnancy is always likely to occur. Whether the unintended pregnancy is because of failed contraceptive method or other reasons, managing the pregnancy will be the most important issue [1, 6–11]. Unintended pregnancies are defined as pregnancies that are unwanted, unplanned or mistimed at the time of conception [12]. According to the latest report about the rate of unintended pregnancy, this issue is still substantially high in developing regions in comparison to developed countries [13–15]. In a recent meta-analysis, the global prevalence of unintended pregnancy has been reported as 48%, with higher prevalence in low-income countries and regions such as Sub-Saharan Africa, West Asia, North Africa and Central and South Asia [16]. In Iran, the prevalence of unintended pregnancy has been reported to be about 30% [17–19].

Reproductive health services are mandatory for these women in order to ensure the health of both mother and child [1]. It has been reported that unintended pregnancies will result in adverse health outcomes for women, their families, and society [20]. Women with unintended pregnancies are more vulnerable to developing suicidal ideation, depression and poor nutrition during pregnancy. They may also experience more physical and psychological violence, unstable family relationships and risk of miscarriage and having low birth weight infants [12, 20]. Some mothers may think of abortion, which is mostly an unsafe induced abortion. Approximately 13% of maternal death is because of such complicated abortions [21–24]. Also, women with unintended pregnancies are more likely to start their pregnancy care late and deny their pregnancy. Such behaviors will increase the risk of developing various pregnancy complications [20, 25–28].

Controversial findings have been reported regarding the possible adverse effects of unintended pregnancy in different countries. Preeclampsia, low birth weight (LBW) and preterm labor are the three most commonly reported complications of unintended pregnancies [29–33]. Considering the importance of the negative consequence of unintended pregnancy and conflicting results, further studies are required to evaluate the relationship between pregnancy intention and potential negative outcomes [34].

Evaluation of these complications in every region by health system managers, policy makers and clinicians, allowing for tailor-made solutions according to the women’s local condition and needs to reduce the risk of such possible complications. The aim of this study was to evaluate the three most common pregnancy complications, including preeclampsia, preterm labor and LBW in women with intended and unintended pregnancies in Sabzevar, northeast Iran in 2019.

## 2. Methods

This prospective follow-up study was conducted in Shahidan Mobini Hospital, Sabzevar, Iran. From December 2018 to September 2019, every married pregnant woman who was referred for prenatal care to the obstetrics-gynecology clinic of hospital was considered to enroll in this study. In order to reduce the possible effect of maternal age on LBW, preterm labor and preeclampsia on the study population, only pregnant women who were between the age of 18 and 35 years old were included in the study [35]. The exclusion criteria were past history of gestational hypertension, gestational diabetes mellitus, fetal malformations, preterm birth, preeclampsia or diagnosis of twin pregnancy. A total of 200 pregnant women, who meet the inclusion criteria, were selected using convenient sampling.

After getting informed consent, in the first visit of women to the clinic a researcher-made questionnaire which included demographic and clinical characteristics of mothers such as age, educational level, gravida, gestational age (weeks) and pregnancy occurrence status (wanted or unwanted from the perspective of both parents) were completed by each of them.

The women were grouped into two groups of unintended or intended pregnancy, according to their response to related questions (100 women in each group). During pre- and postnatal care follow-up visits, all women were regularly and closely assessed in terms of occurrence of three most common pregnancy complications, including preeclampsia, preterm labor and LBW by a maternal-fetal medicine fellowship. All information were recorded.

### 2.1. Theoretical/operational definition

The infant’s birth weight lower than 2500 grams and greater than 1500 grams, regardless of gestational age, were considered as LBW [36]. Preterm labor was considered when the fetus was born before the 37th weeks of gestation [37]. Preeclampsia was considered when the maternal systolic and diastolic blood pressure was higher than 140 mmHg and 90 mmHg respectively, besides presence of proteinuria [38].

### 2.2. Sample size calculation

Estimation of sample size was based on 43% prevalence of unintended pregnancy and its risk factors in previous study [39]. At a level of α = .05 with a power of 0.8, we calculated that it was necessary to enroll 180 eligible women for this study, so in order to allow for a withdrawal rate of 10%, we planned to recruit 200 women (100 women with unintended pregnancies and 100 with intended pregnancies).

### 2.3. Ethical consideration

The protocol of the study was approved by the ethics committee of the Sabzevar University of Medical Sciences, Sabzevar, Iran. The researchers explained the objectives of the study to the participants, and informed consent was obtained from all of them. The participants were informed of their right to refuse or decline participation in the study, at any time and for any reason. They were thoroughly informed and assured that refusing to participate in the study will not affect their current or future medical care.

### 2.4. Statistical analysis

Continuous data were assessed for normality using the Kolmogorov-Smirnov test. Continuous data were presented using mean and standard deviation (SD) while the categorical data were presented using frequency and percentage. Continuous variables were compared between groups using the independent t-test while the categorical variables were compared between groups using the chi-square or Fisher exact test. Associations between maternal pregnancy intention and occurrence of three most common pregnancy complications, including preeclampsia, preterm labor and LBW, were estimated using logistic regression procedures. We estimated the ORs to assess the strength of the associations adjusted for potential confounders and used the 95% CIs to test significance. Data analysis was performed using the statistical package for social sciences (SPSS) software (IBM Inc, Chicago, Il, USA) version 22.0. Level of statistical significance was considered as 0.05.

## 3. Results

Among the study participants, the mean age of women with unintended and intended pregnancy was 29.29 ± 5.01 and 26.89 ± 4.54 years respectively. Demographic characteristics of the pregnant mothers in the study groups are presented in Table 1. Women in the unintended pregnancy group were significantly older (p < 0.001) and had more pregnancies (p < 0.001) (Table 1).

Overall, the most common pregnancy complication was LBW (20.5%) followed by preterm labor (11.5%) and preeclampsia (3%). The prevalence of pregnancy complications among study groups is presented in Table 2. Although the prevalence of three assessed pregnancy complications was higher in the unintended pregnancy group, there was no statistically significant difference in terms of these complications between intended and unintended pregnancy groups (Table 2).

Table 3 shows the results of logistic regression analyses and revealed the relationship between maternal pregnancy intention and the pregnancy complications (preeclampsia, LBW and preterm labor). Women who reported their pregnancies as unintended were 2.11 times more likely to have a baby with LBW than women with intended pregnancies. Also, women with unintended pregnancy was at increased odds of experiencing preterm labor or preeclampsia, even after adjusting for maternal age and gravida.

## 4. Discussion

The present study evaluated the prevalence of three common pregnancy complications among women with intended and unintended pregnancy in Iran. Although the prevalence of these pregnancy complications was higher among the unintended pregnant mothers compared to intended pregnancy group, the difference was not statistically significant. Relative small sample size is a possible explanation for these seemingly contradictory results. However, the results of regression models showed that women with unintended pregnancies were more than twice as likely to have a child with LBW, even after adjusting for maternal age and gravida. The result of recently published study in Bangladesh also revealed that women with unintended pregnancies had three times more likely to have LBW babies [40]. Also, the results of two cohort studies in Iran revealed that there was a significant relationship between unwanted pregnancy and LBW [41–42]. These findings are generally consistent with the results of our study.

Maternal age is one of the most common and important factors in predicting unintended pregnancy. Usually, unintended pregnancies are more prevalent among women who are at the younger and older ends of the childbearing age range. On the other hand, these women are more likely to have LBW infants [43–46]. In line with this, the results of our study revealed that women with unintended pregnancies were significantly older age than women with intended pregnancies.

According to the results of our study, preeclampsia was more prevalent in women with unintended pregnancy, compared to those with intended pregnancy. The results of a study by Adu-Bonsaffoh et al. in Ghana revealed that preeclampsia is more prevalent in unintended pregnancies [29]. Also, the results of another study in India showed that unintended pregnancy is associated with significantly increased risk of preeclampsia and postpartum preeclampsia [47]. It has been previously indicated that advanced maternal age is associated with increased risk and severity of preeclampsia in pregnant women [48–50]. In addition, multiple gestation has been reported as another risk factor for preeclampsia [51–52]. In this study, women with unintended pregnancies were older and had had higher gravidity. After adjusting for these factors, the relationship between unintended pregnancy and preeclampsia was attenuated, but still elevated.

A recent study in Iran indicated that neither preeclampsia nor LBW and preterm labor were related to unintended pregnancy and reported the higher rate of cesarean section and inappropriate weight gain during pregnancy, as the possible outcomes of unintended pregnancy [30]. Unfortunately, evaluating these two factors was beyond the aims of this study, which focused on the three most common pregnancy complications between unintended and intended pregnancy.

The prospective nature of this study and regular follow-up of the women are strengths of the study, however, using larger number of participants would be useful to draw a more concrete conclusion about the results. Lack of information regarding women’s pre-conception, antenatal, delivery and postnatal behaviors, as potential confounders, is another limitation that could be considered in further studies.

## 5. Conclusion

In conclusion, the findings of this study indicated that unintended pregnancy can be a risk factor for pregnancy complications including preeclampsia and LBW. Therefore, tailor-made interventions are required to be developed and implemented for reducing unintended pregnancies and prevent their potential common complications.

## Figures and Tables

**Table 1:** Demographic and clinical characteristics of all pregnant women.

Variables		Unintended pregnancy (n =100)	Intended pregnancy (n =100)	P-value
Education level	Primary	79 (79%)	69 (69%)	0.10
	Secondary	21 (21%)	31 (31%)	
Age (years)		29.29 ± 5.01	26.89 ± 4.54	<0.001^a^
Gravida		2.60 ± 0.96	1.88 ± 0.86	<0.001^a^
Gestational age at delivery (weeks)		38.97 ± 1.4	39.15 ± 17.1	0.50

a Significant at α =0.01.

**Table 2:** Comparison of pregnancy complication in women with intended and unintended pregnancy.

Complications	Unintended pregnancy (n =100)		Intended pregnancy (n =100)		Total	P-value ᵻ
	
	Yes	No	Yes	No		
Preeclampsia	5 (5%)	95 (95%)	1 (1%)	99 (99%)	6 (3%)	0.21
LBW	25 (25%)	75 (75%)	16 (16%)	84 (84%)	41 (20.5%)	0.16
Preterm labor	12 (12%)	88 (88%)	11 (11%)	89 (89%)	23 (11.5%)	0.50

LBW =Low Birth Weight;

ᵻ Fisher exact test was used for the analysis.

**Table 3:** Association between maternal pregnancy intention and pregnancy complications^a^.

Variables	Crude OR	95% CI	Adjusted OR^b^	95% CI
Preeclampsia	1.71	0.95–3.64	1.18	0.67–2.31
LBW	2.53	0.8–4.51	2.11	0.54–3.35
Preterm labor	1.36	0.63–1.47	1.06	0.53–1.69

OR, Odds ratio; CI, Confidence interval.

a Reference group is women with intended pregnancies.

b All outcomes adjusted for mothers’ age and gravida.
